# Effect of transcutaneous electrical acupoint stimulation on the quality of postoperative recovery: a meta-analysis

**DOI:** 10.1186/s12871-024-02483-z

**Published:** 2024-03-19

**Authors:** Meng Zhang, Huanhuan Zhang, Pan Li, Jianli Li

**Affiliations:** Department of Anesthesia, Hebei General Hospital, Shijiazhuang, 050051 China

**Keywords:** Transcutaneous electrical acupoint stimulation, Quality of recovery, General anesthesia, Meta-analysis, Randomized controlled trial

## Abstract

**Background:**

The purpose of the present study was to systematically delve into the efficacy and safety of transcutaneous electrical acupoint stimulation (TEAS) on the quality of recovery after general anesthesia.

**Methods:**

Randomized controlled trials related to TEAS improving postoperative recovery quality were searched in Cochrane Library, Web of Science, Embase, PubMed, CNKI, VIP, Wanfang and Chinese biomedical database from the inception of each database to June 2023. After literature screening and data extraction, Stata15 software was employed for meta-analysis, and the quality of the included literature was evaluated utilizing ROB2.

**Results:**

The study included 10 articles involving 2,383 patients in total. The meta-analysis results unveiled that TEAS could improve 24-hour and 48-hour postoperative QoR-40 scores as well as 24-hour postoperative QoR-40 dimension scores [WMD = 8.52, 95%CI (5.12, 11.91), *P* < 0.001; WMD = 1.99, 95%CI (0.91, 3.07), *P* < 0.001], emotional state [WMD = 1.38, 95%CI (0.66, 2.09), *P* < 0.001], physical comfort [WMD = 2.99, 95%CI (1.59, 4.39), *P* < 0.001], psychological support [WMD = 0.63, 95%CI (0.36, 0.90), *P* < 0.001], and physical independence [WMD = 0.76, 95%CI (0.22, 1.30), *P* = 0.006]; pain [WMD = 1.81, 95%CI (0.87, 2.75), *P* < 0.001]; decrease 24-hour postoperative VAS pain scores [WMD = -0.84, 95%CI (-1.45, -0.23), *P* = 0.007] and the incidence of postoperative nausea and vomiting [RR = 0.88, 95%CI (0.81, 0.97), *P* = 0.006; RR = 0.62, 95%CI (0.52, 0.73), *P* < 0.001].

**Conclusion:**

TEAS can improve postoperative QoR-40 scores and the quality of recovery, relieve pain, and decrease the incidence of nausea and vomiting after surgery in patients who underwent general anesthesia.

**Trial registration:**

CRD42023433959.

**Supplementary Information:**

The online version contains supplementary material available at 10.1186/s12871-024-02483-z.

## Background

With the advancement of surgical and anesthesia techniques, the quality of recovery (QoR) after surgery has attracted clinical attention. The assessment of QoR has important clinical significance for patient prognosis and potential for research application, and thus evaluating the QoR of patients is imperative. In a systematic review comparing the assessment scales commonly used in anesthesia studies to evaluate postoperative quality of recovery, the QoR-40 score was the most commonly used, with a usage rate of 42.6% [1]. Postoperative recovery is an intricate process affected by various factors including cognitive function, emotional state, pain, stress response and physical dysfunction [2]. The QoR-40 questionnaire consisting of 40 questions comprehensively measures the quality of postoperative recovery from five dimensions, and its validity has been confirmed in previous studies [3].

Transcutaneous electrical acupoint stimulation (TEAS) is a new type of non-invasive therapy that combines traditional acupuncture with transcutaneous electrical nerve stimulation, with the advantages of ease of use and non-invasiveness. The benefits and potential risks of TEAS for improving QoR-40 are still controversial. The study by Yu et al. [4] found that TEAS improved QoR-40 scores and reduced nausea and vomiting after surgery, while Liang et al. [5] concluded that TEAS improved postoperative QoR-40 scores but did not decrease the incidence of postoperative nausea and vomiting. The research results of Lv et al. [6] revealed higher QoR-40 scores in the TEAS group at 24 and 48 h after surgery compared to the control group, yet Mi et al. ^[7]^ found that TEAS could improve the QoR-40 score at 24 h after surgery, but not at 48 h postoperatively. Mi et al. [7] also concluded that TEAS could improve the overall quality of recovery in the following five dimensions: physical comfort, emotional state, self-care ability, psychological support, and pain. However, Pan et al. [8] observed no significant difference in terms of psychological support and self-care ability between the TEAS and the control groups. These controversial conclusions need to be integrated by meta-analysis.

Currently, there is no available meta-analysis on TEAS’s effect on the postoperative quality of recovery. Therefore, the present study aimed to investigate the efficacy and safety of perioperative TEAS in improving the quality of postoperative recovery by combining relevant literature using meta-analysis.

## Methods

We have registered the present study with PROSPERO under registration number CRD42023433959.

### Literature search

A comprehensive search was conducted in Cochrane Library, Web of Science, Embase, PubMed, VIP, Wanfang, CNKI, and Chinese biomedical databases for randomized controlled trials on TEAS’s effect on the quality of recovery after general anesthesia published from the inception of each database to June 2023 without restricting the language. The search strategy included: the study population of patients undergoing surgery under general anesthesia; intervention of “transcutaneous acupoint electrical stimulation” or “electroacupuncture”; the control group of no TEAS or sham TEAS; outcome measure of QoR-40, and study type of randomized controlled trial. Table 1 shows the search strategy in PubMed.

**Table 1:** Search strategy in PubMed

#1 Transcutaneous electrical acupoint stimulation[Title/Abstract]
#2 Transcutaneous acupoint electrical stimulation[Title/Abstract]
#3 acustimulation[Title/Abstract]
#4 TEAS[Title/Abstract]
#5 #1 OR #2 OR #3 OR #4
#6 Quality of recovery[Title/Abstract]
#7 Quality[Title/Abstract]
#8 Recovery[Title/Abstract]
#9 #7 AND #8
#10 #6 OR #9
#11 #5 AND #10

### Inclusion and exclusion criteria

The inclusion criteria are as follows: (1) Research design: randomized controlled trial (RCT); (2) Study subjects: patients undergoing surgery with general anesthesia; (3) Interventions: the experimental group adopted transcutaneous electrical acupoint stimulation, and acupoint selection and transcutaneous acupoint stimulation device were not differentiated; the control group used sham TEAS or no intervention measures or other targeted intervention measures; (4) Outcome measures: the QoR-40 score after surgery was used as the primary outcome measure. The QoR-40 questionnaire includes five dimensions of physical comfort (12 items), psychological support (7 items), emotional state (9 items), pain (7 items) and Physical independence (5 items), with each item rated 5 points. QoR-40 scores range from 40 to 200, with higher scores suggesting a better quality of recovery [9]. Secondary indicators were postoperative VAS scores, as well as incidence of nausea and vomiting after surgery. The exclusion criteria included: (1) Animal experiments, conference data, case reports, systematic evaluations and reviews; (2) Duplicate content, incorrect or incomplete research data, or inaccessible literature; (3) The QoR-40 score was not used in the literature.

### Literature screening and data extraction

Endnote was employed to eliminate duplicates from the retrieved literature, and two independent researchers were assigned to read article titles and abstracts to exclude literature that was not relevant to the research topic. After a thorough reading of the literature, RCTs meeting the study requirements were screened in accordance with the inclusion and exclusion criteria. The valid data were extracted from the included literature using a unified data table, and the extracted information mainly encompassed publication year, the first author, sample size, age, sex, the information of blinding, type of surgery, interventions, acupoint selection and intervention time. The whole process of literature screening was carried out separately by two researchers, and a third-party reviewer participated in the discussion when there was any disagreement on a certain literature.

### Literature quality assessment

The quality of the included studies was evaluated based on ROB2 by two researchers (ZM, Z-HH) [10]. The specific contents are as follows: (1) bias during randomization; (2) bias in deviating from established interventions; (3) bias in outcome measurement; (4) bias in selective reporting of outcomes; and (5) bias in missing outcome data. According to different research purposes, the bias in deviating from established interventions was categorized into one to study the effect of intervention distribution, and the other to study the effect of intervention compliance. Risk levels were categorized into three levels of high risk, low risk, and unclear. Literature that was difficult to evaluate was discussed and decided jointly with a third party.

### Statistical Processing

Stata 15.1 software was employed for meta-analysis. Dichotomous and continuous variables were represented by risk ratio (RR) and weighted mean difference (WMD), respectively, with 95% confidence interval (CI). *P* < 0.05 was considered to indicate a statistically significant difference. In view of the clinical diversity and high methodological heterogeneity between studies, we conducted the analysis using a random-efects model. Subgroup analysis or sensitivity analysis was performed to investigate the influence of individual studies on the stability of meta-analysis results.

## Results

### Literature retrieval

**Fig. 1:**
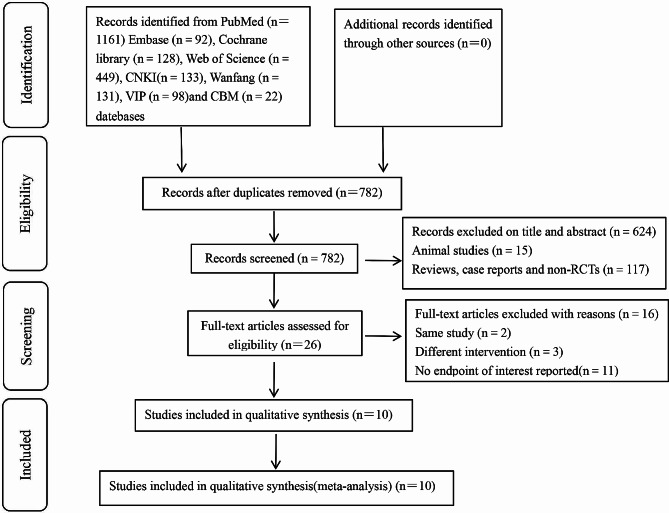
Flowchart of literature inclusion

The initial search of the databases yielded 1,161 articles and after removing 379 of them, we obtained 782 articles. By reviewing the titles and abstracts, 756 articles were excluded for not meeting the requirements of the study, and after reading the remaining 26 papers in full text, 16 with repeated publication and inconsistent intervention time and outcome measures were excluded. Finally, 10 articles [4–9, 11–14] conforming to the research content were included, and the screening process is shown in Fig. 1.

### Basic characteristics of included studies

This study included 10 articles published from 2015 to 2023, involving 2,383 patients, with 1,189 in the observation group and another 1,194 in the control group. The observation group received TEAS in 10 articles [4–9, 11–14], while the control group received sham TEAS in 9 papers [4–7, 9, 11–14] and no TEAS in one paper [8]. In the SHAM TEAS group, the patients were connected to the apparatus in the same acupoints, but electronic stimulation was not applied. The surgical types were gynecological laparoscopic surgery, laparoscopic non-gastrointestinal surgery, laparoscopic cholecystectomy, radical mastectomy, transurethral resection of the prostate, supratentorial surgery and thyroidectomy. The commonly selected acupoints in the 10 papers are PC6, ST36 and LI4, as presented in Table 2.

**Table 2:** Basic characteristics of included randomized controlled trials

Author year	Sample size (T/C)	Age yr (T/C)	Sex (F/M)	Information of blinding	Types of surgery	Intervention Group	Control Group	Acupoints	Intervention time	Electrical frequency
Lv et al. 2022	103(52/51)	37. 17 ± 10. 59 34. 63 ± 9.03	52/0 51/0	yes	laparoscopic non-gastrointestinal surgery	TEAS	sham TEAS	PC6, ST36	15 min after the end of anesthesia,from 7 am to 12 am on the first day after surgery	2/100 HZ
Jin et al. 2020	61(30/31)	48.20 ± 6.90 50.90 ± 7.10	30/0 31/0	No described	radical mastectomy	TEAS	sham TEAS	LI4, PC6, ST36, SP6	30 min before the induction of anesthesia,until the end of anesthesia	2/100 HZ
Mi et al. 2018	100(50/50)	44 ± 6 45 ± 8	22/28 24/26	No described	laparoscopic cholecystectomy	TEAS	sham TEAS	LI4, PC6, ST36 and the non-acupoint 2 cun outboard from ST36	30 min before the induction of anesthesia,until the end of anesthesia	2/100 HZ
Pan et al. 2023	105(52/53)	42.2 ± 5.5 43.2 ± 6.3	52/0 53/0	No described	laparoscopic myomectomy	TEAS	No TEAS	LI4, PC6, ST36, SP6	for 30 min before the operation and lasting until the end of anesthesia	2/100 HZ
Gao et al. 2022	1655 (827/828)	39.0(31.0,46.0) 39.0(31.0,46.0)	803/24 803/25	yes	laparoscopic non-gastrointestinal surgery	TEAS	sham TEAS	PC6 and ST36	in the PACU when recovered from anesthesia on the same surgical day and on the next morning of surgical ward for 30 min	2/10 Hz
Liang et al. 2021	70(35/35)	70.8 ± 6.5 60.9 ± 6.1	0/35 0/35	yes	TURP	TEAS	sham TEAS	CV7, CV6, CV5, CV4, CV3, BL32, BL33, BL34	for 30 min	2/100 HZ
Yu et al. 2020	60(30/30)	45.9 ± 17.5 48.5 ± 16.2	30/0 30/0	yes	gynecological laparoscopic surgery	TEAS	sham TEAS	GV20, EX-HN3, ST36, PC6	for 30 min before anesthesia	2/100 Hz
Bai et al. 2018	75(37/38)	66.0 ± 3.0 65.1 ± 3.4	19/18 22/16	yes	supratentorial craniotomy	TEAS	sham TEAS	LI4, PC6, LU7, LU5, LI18, ST9	30 min before the induction of anesthesia,until 5 min before the end of anesthesia	2/10 Hz
Chen et al. 2015	83(41/42)	37.5 ± 8.5 40.2 ± 7.8	41/0 42/0	yes	thyroidectomy	TEAS	sham TEAS	LI4, PC6	30 min before the induction of anesthesia	2/10 Hz
Yao et al. 2015	71(35/36)	34.2 ± 7.2 35.6 ± 8.7	35/0 36/0	yes	gynecological laparoscopic surgery	TEAS	sham TEAS	LI4, PC6, ST36, SP6	30 min before the induction of anesthesia	2/10 Hz

RCT: randomized controlled trial; T: the TEAS group; C: the control group; TEAS: transcutaneous electrical acupoint stimulation; acupoint: Neiguan PC6, Zusanli ST36, Hegu LI4, Sanyinjiao SP6, Yinjiao CV7, Qihai CV6, Shimen CV5, Guanyuan CV4, Zhongji CV3, Ciliao BL32, Zhongliao BL33.Xiaoliao BL34, Baihui GV20, Yintang EX-HN3, Lieque LU7, Chize LU5, Futu LI18, Renying ST9.

### Quality evaluation of included studies

**Fig. 2:**
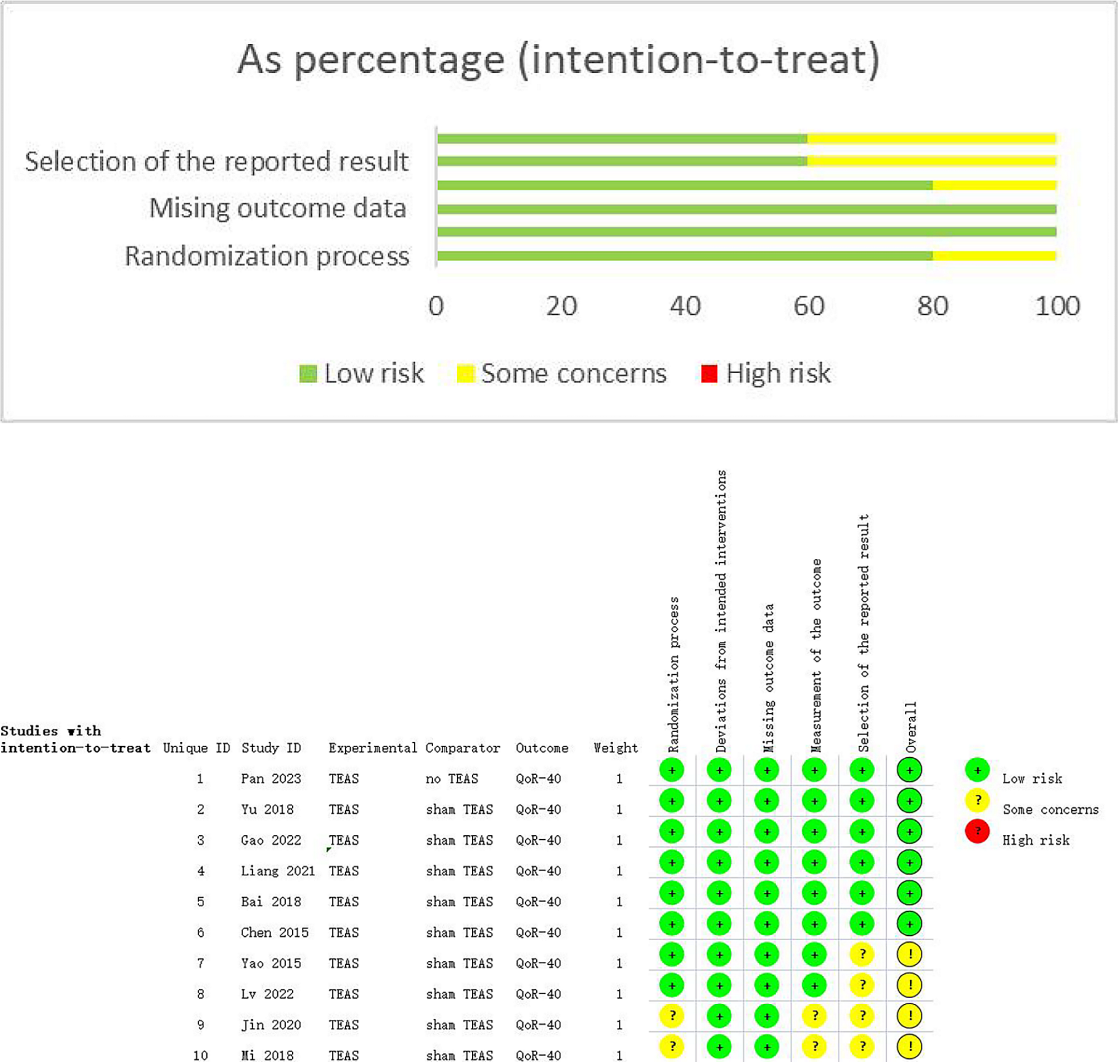
Risk of bias assessment of included studies

Figure 2 shows the risk of bias assessment results of the 10 included studies. The risk of bias in six studies [4, 5, 8, 9, 12, 13] was low in all five domains and they provided the most comprehensive data. Four RCTs [6, 7, 11, 14] had a moderate risk of bias, most commonly owing to an absence of reference to the specific randomization process and whether the outcome data was analyzed with reference to a pre-specified analysis plan.

### Main results

#### 24-hour postoperative QoR-40 score

**Fig. 3:**
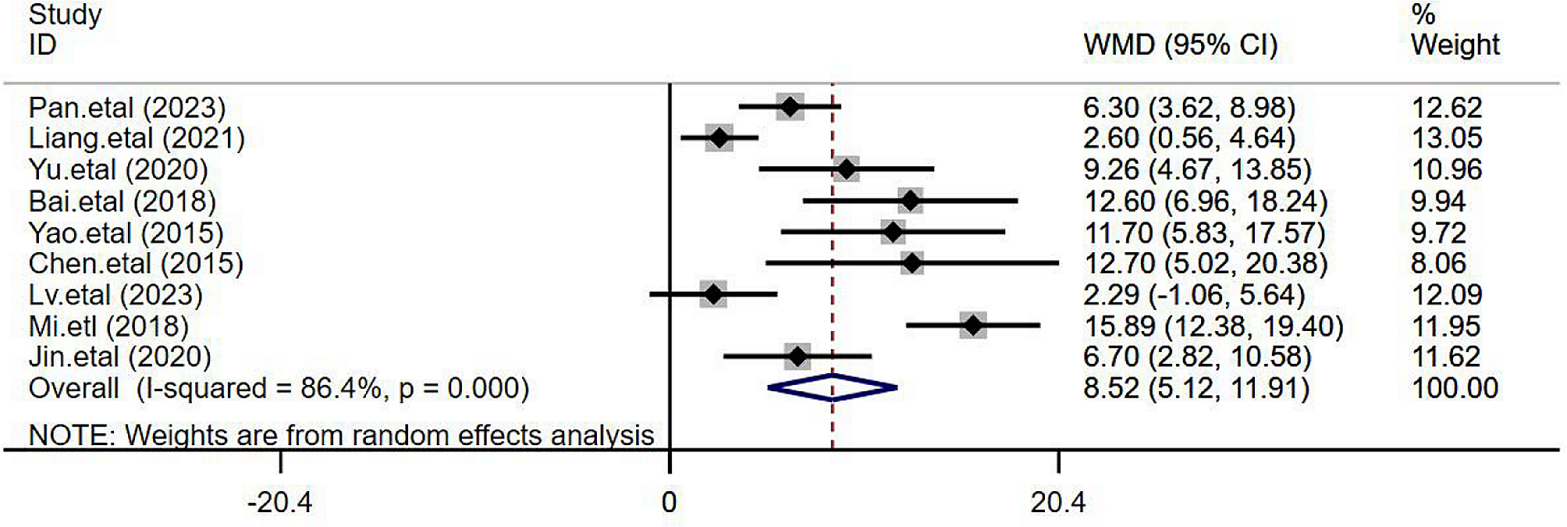
Forest plot comparing the 24-hour postoperative QoR-40 score between the two groups

The 24-hour postoperative QoR-40 score was adopted in nine papers [4–9, 11, 12, 14]to assess the improvement of TEAS on quality of recovery in patients who received general anesthesia. With 362 cases and 366 cases in the TEAS and the control groups respectively, significant heterogeneity was found among studies (I^2^ = 86.4%, *P* < 0.0001), and thus the random-effects model was adopted. The results revealed that compared to the control group, the TEAS group could significantly improve the 24-hour postoperative QoR-40 score (WMD = 8.52, 95%CI 5.12 to 11.91, *P* < 0.001), as presented in Fig. 3.

### 48-hour postoperative QoR-40 score

**Fig. 4:**
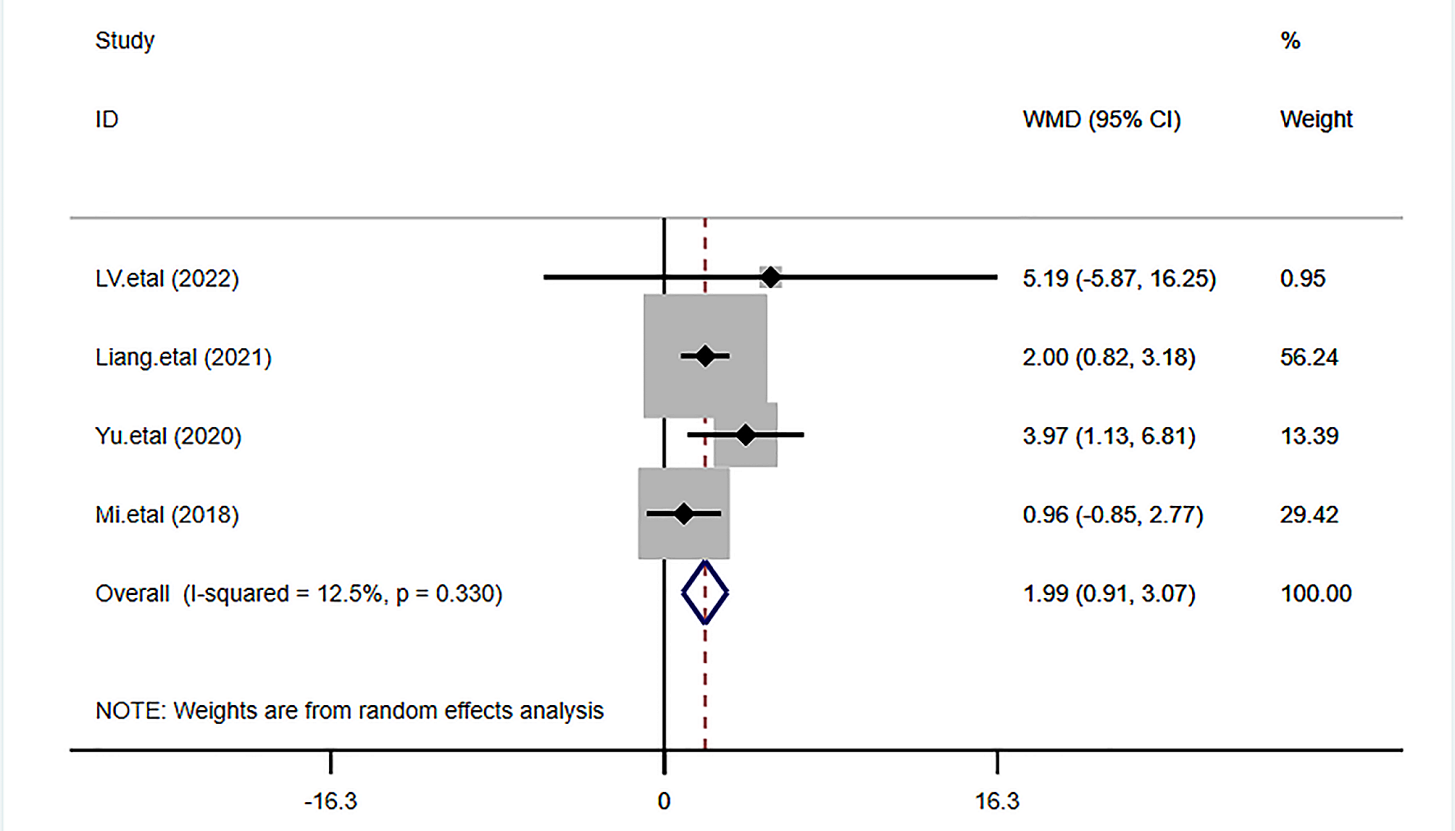
Forest plot comparing the 48-hour postoperative QoR-40 score between the two groups

Four studies [4–7] reported 48-hour postoperative QoR-40 scores, including 167 cases and 166 cases in the TEAS and the control groups respectively, and we used the random-effects model. We found significantly higher 48-hour postoperative QoR-40 scores in the TEAS group than in the control group, which was statistically significant (MD = 1.99, 95%CI 0.91–3.07, *P* < 0.001) (Fig. 4).

### 24-hour postoperative QoR-40 dimension scores

**Fig. 5:**
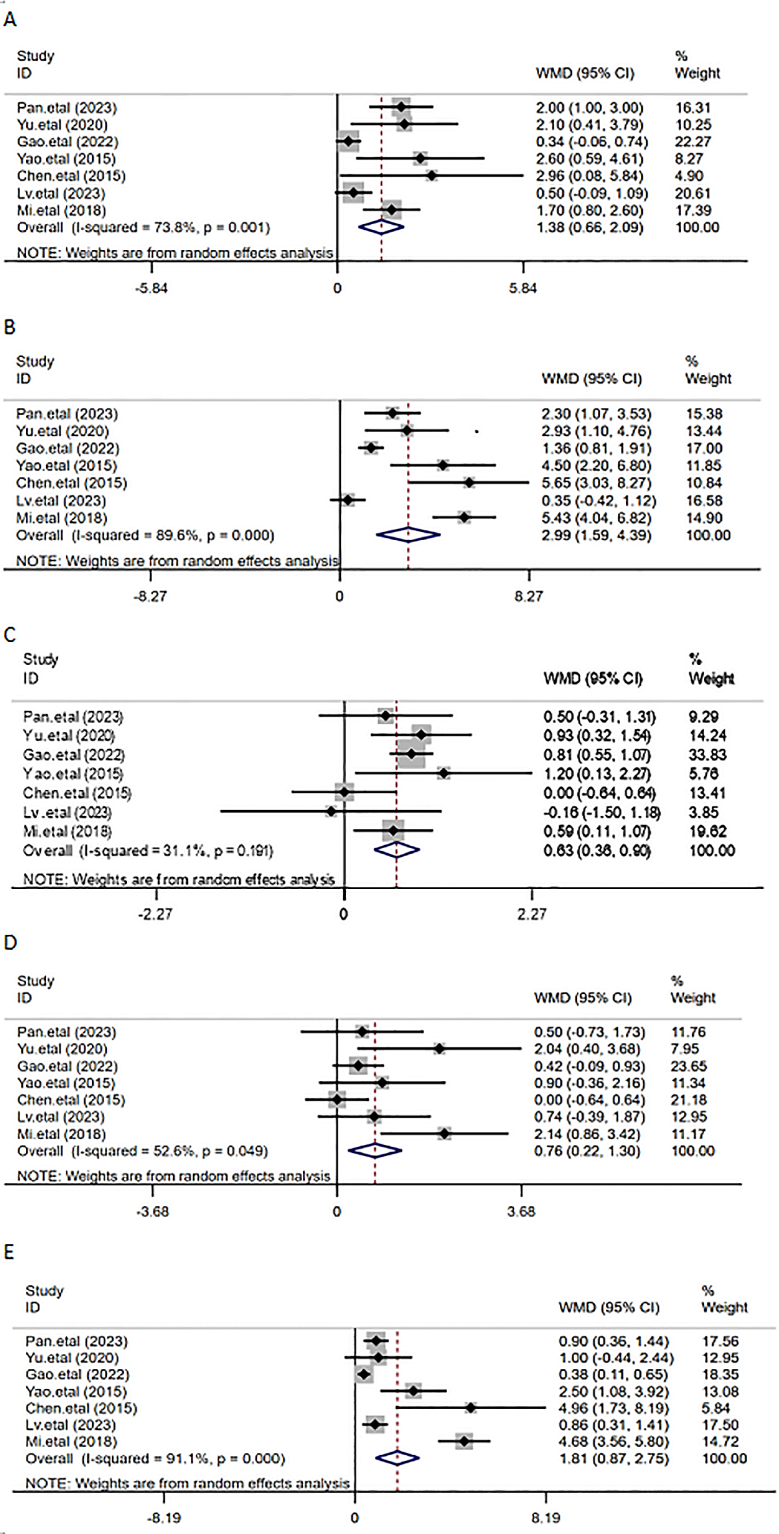
Meta-analysis of TEAS on QoR-40 dimension scores. (**A**) Emotional state; (**B**) Physical comfort; (**C**) Psychological support; (**D**) Physical independence; (**E**) Pain

Seven studies [4, 6–9, 11, 13] evaluated the improvement of TEAS on postoperative quality of recovery in those who underwent general anesthesia from various dimensions of QoR-40. The meta-analysis results suggested that in comparison with the control group, the five indicators of the 24-hour postoperative QoR-40 score, namely physical comfort, physical independence, emotional state, and pain, were significantly higher in the TEAS group [for emotional state, WMD = 1.38, 95% CI ( 0.66,2.09), *P* < 0.001; for physical comfort, WMD = 2.99, 95% CI (1.59,4.39), *p* < 0.001; for psychological support, WMD = 0.63, 95%CI (0.36, 090), *P* < 0.001; for physical independence, WMD = 0.76, 95%CI (0.22,1.30), *P* = 0.006; for pain, WMD = 1.81, 95%CI (0.87,2.75), *P* < 0.001]. The random-effect model was employed, revealing statistically significant differences, as shown in Fig. 5.

### VAS pain score

**Fig. 6:**
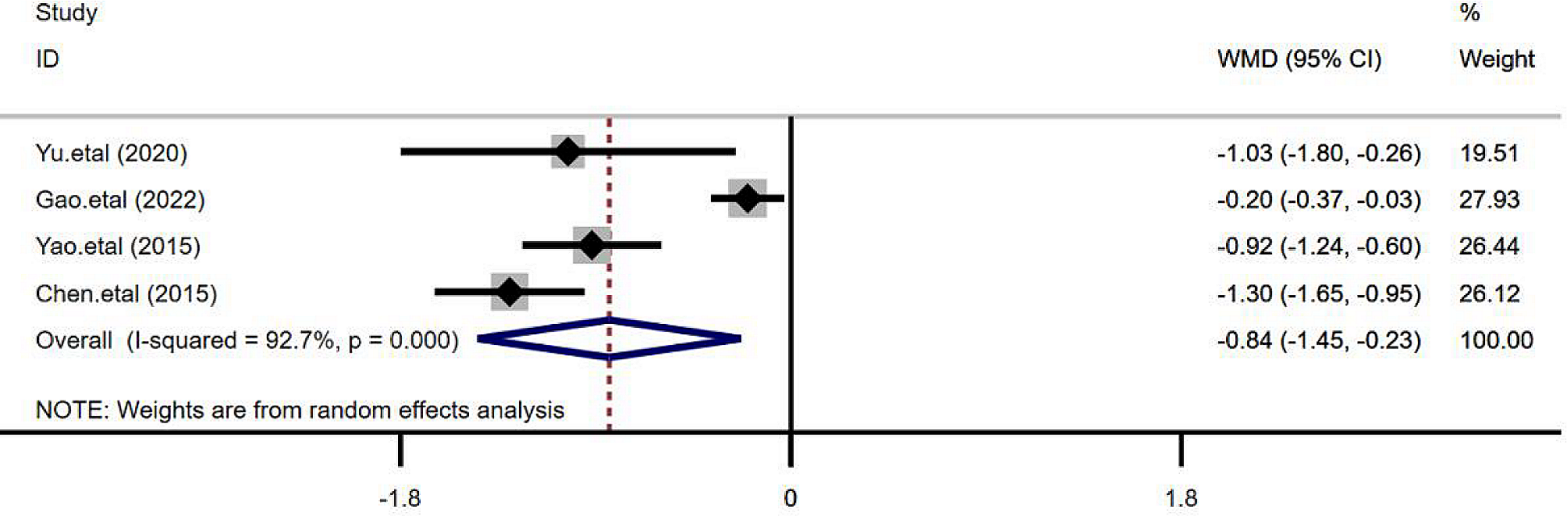
Forest plot comparing postoperative VAS scores between the two groups

**Fig. 7:**
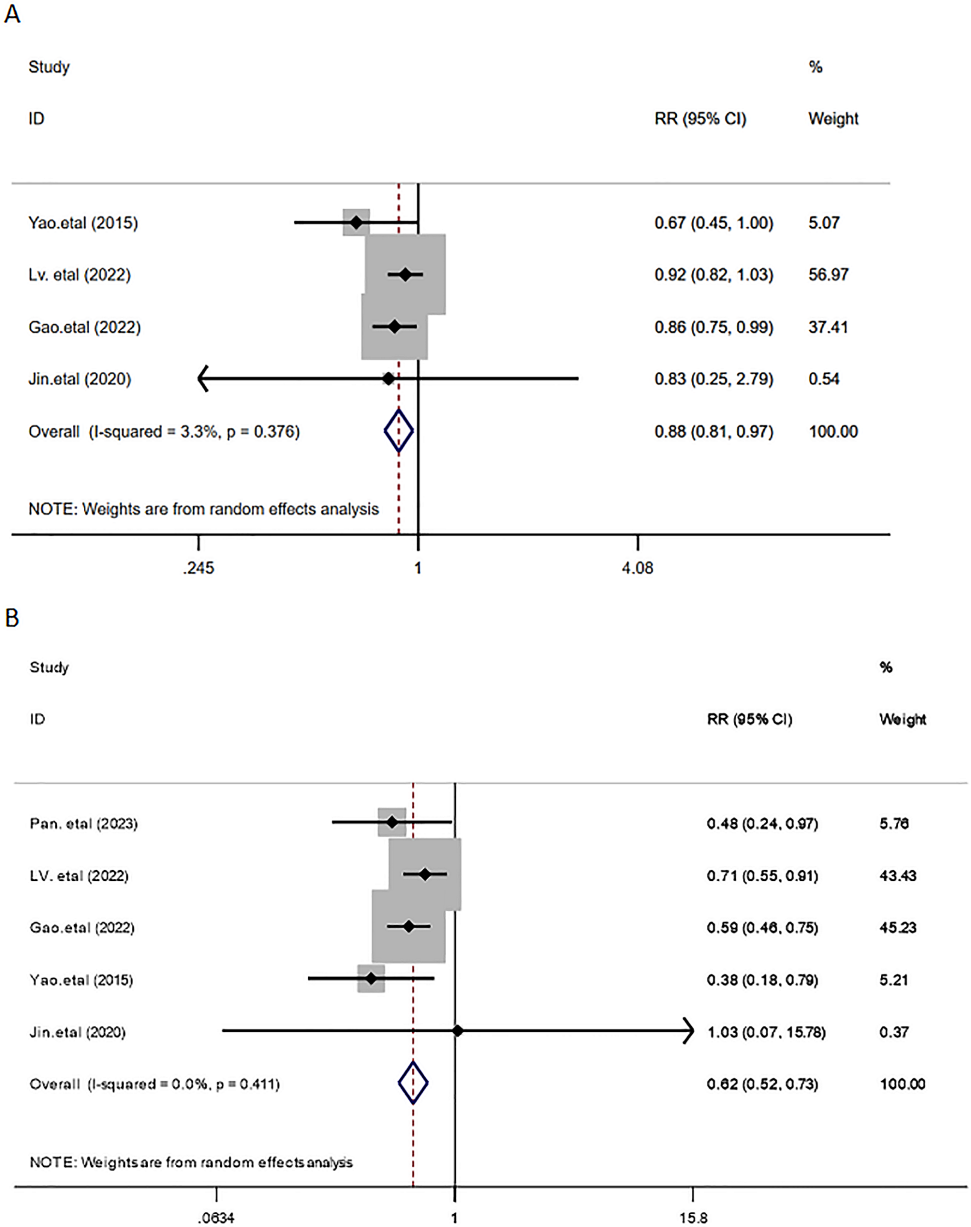
Meta-analysis of TEAS for incidence of postoperative nausea or vomiting. (**A**) Postoperative nausea. (**B**) Postoperative vomiting

Four studies [4, 9, 11, 13] used VAS pain score to evaluate the pain degree 24 h after surgery, including 993 cases and 936 cases in the TEAS and the control groups respectively. Our meta-analysis results using a random-effects model demonstrated that the 24-hour postoperative VAS pain score in the TEAS group was significantly lower compared to the control group [WMD = -0.84, 95%CI (-1.45, -0.23), *P* = 0.007], and the difference was found to be statistically significant, as shown in Fig. 6. Four papers [6, 11, 13, 14] reported the occurrence of postoperative nausea, including 944 cases and 946 cases in the TEAS and the control groups respectively. The meta-analysis indicated a significantly lower incidence of postoperative nausea in the TEAS group than that in the control group [RR = 0.88, 95%CI (0.81, 0.97), *P* = 0.006], with a significant difference. Five papers [6, 8, 11, 13, 14] reported the occurrence of postoperative vomiting, including 996 cases and 999 cases in the TEAS and the control groups respectively. The incidence of postoperative vomiting in the TEAS group was shown to be significantly lower than that in the control group [RR = 0.62, 95%CI (0.52, 0.73), *P* < 0.001], and the difference was significant (Fig. 7).

### Subgroup analysis results

**Fig. 8:**
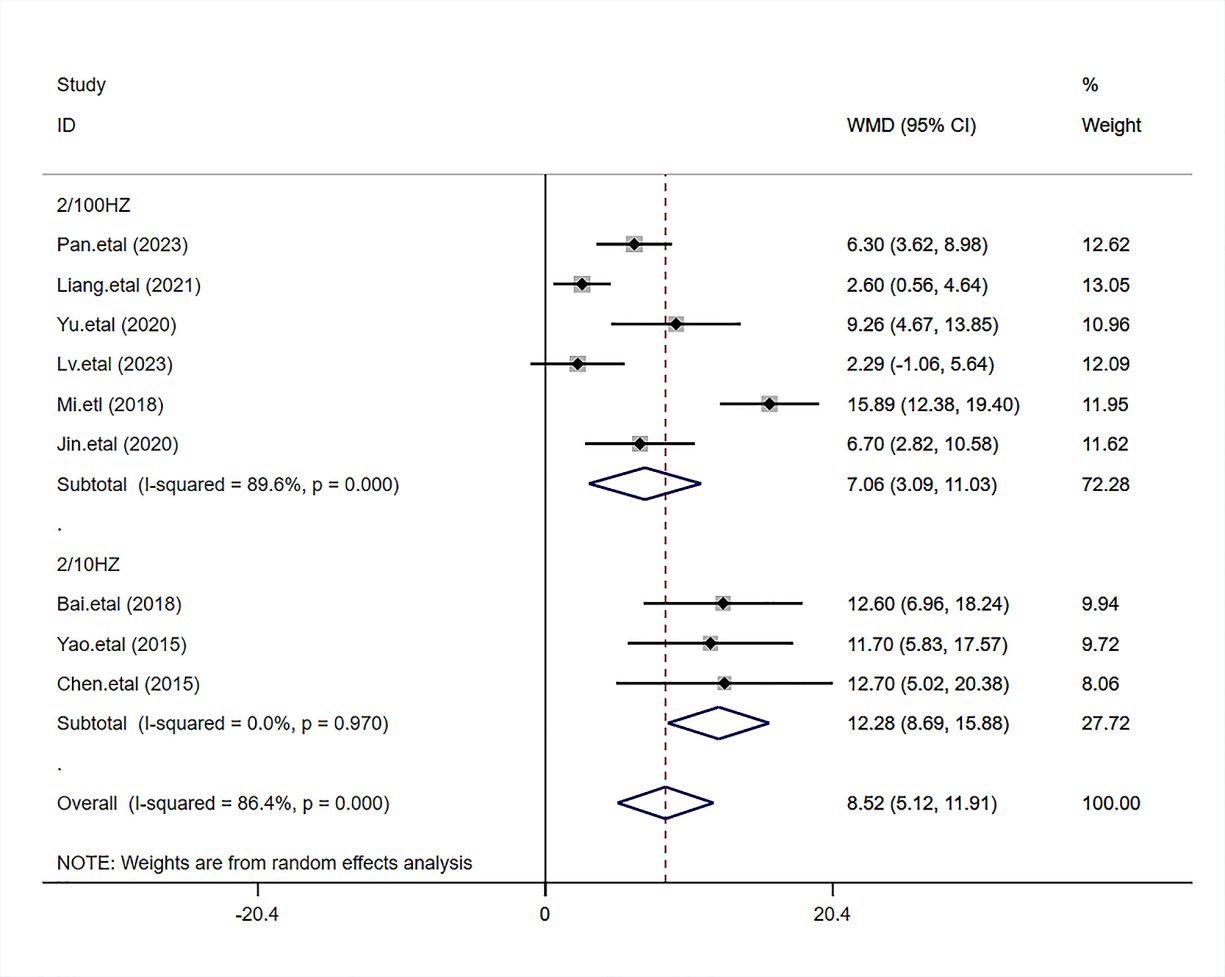
Subgroup analysis based on different frequencies

Our subgroup analysis, basing on the different frequencies of stimulation, indicated that in comparison with the control group, two types of frequencies could significantly improve the 24-hour postoperative QoR-40 score in the TEAS group [for 2/100Hz, WMD = 7.06,0.95% CI (3.09,11.03)*P* < 0.001;for 2/10Hz, WMD = 12.28,95% CI (8.69,15.88),*P* < 0.001],(Fig. 8).

### Safety evaluation

Skin infection, redness and swelling, ecchymosis and rupture of the skin at the site of electrical stimulation were not reported in any of the 10 included papers. None of the subjects in the literature had any significant abnormal reactions during treatment (e.g., pain intolerance due to electrical stimulation, etc.).

## Discussion

Postoperative quality of recovery after anesthesia is critical in assessing the success of surgery, and also an important indicator to judge the initial health status of patients after the operation. As interest in quality of recovery grows and the concept of holistic rehabilitation becomes more important, the field of anesthesia is actively developing multiple methods for assessing this indicator, and one of the most frequently used tools is the QoR-40 questionnaire developed by Myles et al. [15] QoR-40 is specifically designed to assess patients’ early postoperative recovery after different types of surgery, and its good reliability and effectiveness have been widely verified. TEAS is non-invasive and safe, with good effects in reducing intraoperative anesthetic drug consumption, improving postoperative pain, alleviating postoperative nausea and vomiting, and promoting postoperative recovery ^[4–9]^. Although clinical evidence suggests that TEAS can promote postoperative recovery in children and adults, its benefits in improving postoperative QoR-40 and potential risks remain controversial. Hence, the present meta-analysis was conducted to investigate the efficacy and safety of TEAS in improving postoperative quality of recovery.

The meta-analysis results of this study demonstrated significantly higher QoR-40 scores 24 and 48 h after surgery as well as 24-hour postoperative QoR-40 dimension scores in patients receiving TEAS compared with the control group, suggesting that TEAS can improve quality of recovery in patients receiving general anesthesia. The reasons for TEAS to improve quality of recovery may include the following: (1) The stress response caused by surgical trauma can last for days or weeks, resulting in poor prognosis of patients [16]. TEAS can reduce the level of perioperative stress in patients, inhibit the release of stress factors, and regulate the immune function of the body [17, 18], thus improving the postoperative quality of recovery; (2) TEAS can reduce perioperative opioid use and decrease the incidence of PONV [19]. In addition, TEAS can also increase the content of gastric actin, accelerate the recovery of gastrointestinal function, improve postoperative nausea and vomiting [20], improve patient satisfaction, and thus accelerate the recovery after surgery; (3) TEAS can activate different neurotransmitters (enkephalin, endorphin, and dynorphin) to exert a synergistic analgesic effect to reduce patients’ postoperative pain, alleviate patients’ nervousness and anxiety [21, 22], increase physical comfort, and thus contribute to rapid perioperative recovery. Dysfunction of the hypothalamic-pituitary-adrenal (HPA) axis has been shown to be a major cause of psycho-behavioral symptoms such as pain sensitivity, depression, and fatigue [23]. Yu et al. [4] proposed that TEAS could improve the quality of recovery after general anesthesia, possibly related to the regulation of the HPA axis function by acupuncture and electroacupuncture.

It has now been demonstrated that TEAS improves the quality of recovery and increases QoR-40 scores. The results of Pan et al. [8] indicated that TEAS improved postoperative 24-hour QoR-40 scores greatly in patients who received laparoscopic myomectomy. Another study showed that the application of TEAS treatment improved quality of recovery and QoR-40 scores in those undergoing transurethral resection of the prostate [5]. The present meta-analysis of 10 RCTs revealed that TEAS significantly improved the postoperative QoR-40 total score and dimension scores, in addition to reduced pain and decreased incidence of nausea and vomiting after surgery. The most frequently selected acupoints in the 10 publications were PC6 (90% 9/10), ST36 (70% 7/10), and LI4 (60% 6/10). LI4 was proved to be associated with analgesic and sedative effect [24], ST36 was stimulated to promote the recovery of gastrointestinal function [8] and PC6 was suggested to mitigate PONV [25]. The results of Liang et al. [5] showed that TEAS improved postoperative QoR-40 scores but did not decrease the incidence of postoperative nausea and vomiting, which might due to the absence of PC6 stimulation. A meta-analysis showed that the use of three acupoints, LI4, PC6 and ST36, for laparoscopic analgesia had a synergistic effect and significantly reduced postoperative pain [26]. The improvement of postoperative quality of recovery may be associated with the reduction of postoperative pain and the decrease of the occurrence of adverse reactions, i.e., nausea and vomiting [8]. Therefore, many studies have chosen these acupoints to improve the quality of recovery after operation.

Wang et al. [27] found that the application of TEAS could relieve postoperative pain. Our study results unveiled that the 24-hour postoperative VAS scores of patients receiving TEAS were significantly lower than those of control patients. However, there was significant heterogeneity (I^2^ = 92.7%, *P* < 0.0001), and sensitivity analysis performed on the included studies showed that the heterogeneity among the studies became smaller after excluding one study [13]. The possible reason may be that the time of implementation of TEAS in this study was after awakening from anesthesia and on the morning of the first postoperative day, whereas in other studies, TEAS was administered before anesthesia induction. Our study results indicate TEAS can reduce the incidence of postoperative nausea and vomiting, which is consistent with previous studies [28, 29]. The occurrence of postoperative nausea was reported four papers and five reported postoperative vomiting, suggesting the reliability of the conclusion, which further demonstrates the beneficial effect of TEAS in improving postoperative quality of recovery in patients undergoing general anesthesia.

### Limitations

The limitations of this study are as follows. First, there is certain heterogeneity in the results of 24-hour QoR-40 total score and dimension scores as well as 24-hour postoperative VAS score. Postoperative quality of recovery and pain are subjective indicators that are highly dependent on the subjective evaluation of clinical evaluators and are difficult to measure by some objective assessment tools due to the subjective impact of patients [30], which may ultimately result in a high degree of heterogeneity. We performed a sensitivity analysis of 24-hour QoR-40 total score and dimension scores and found no significant changes in the study results, indicating the stability of this study as well as the reliability of the research results. A sensitivity analysis of the postoperative 24-hour VAS score was performed, and the heterogeneity among studies became smaller after excluding one article [9], possibly due to the different implementation times of TEAS between the excluded literature and other studies. Second, only QoR-40 in patients in the early postoperative period was assessed in the included literature, without effectively assessing the long-term efficacy of TEAS in improving postoperative quality of recovery. Third,the majority of patients were female in our study since some female surgeries were included, which might lead to an unbalanced ratio between males and females. Since only 10 articles meet the requirements after screening, to further verify the effectiveness of TEAS in improving postoperative quality of recovery, more RCTs with large samples and high quality are still required for comprehensive evaluation in the future, thereby providing sufficient evidence-based medical evidence for clinical practice.

## Conclusion

In summary, based on existing clinical studies, TEAS can improve the quality of recovery of patients who underwent general anesthesia, relieve pain, and reduce the incidence of postoperative nausea and vomiting. Meanwhile, more RCTs with large samples and high quality are required to confirm the current findings.

### Electronic supplementary material

Below is the link to the electronic supplementary material.

Supplementary Material 1

Supplementary Material 2

Supplementary Material 3

## Data Availability

All data generated or analysed during this study are included in this article and its supplementary information files.
