# Supplementary material for: Effect of transcutaneous electrical acupoint stimulation on the quality of postoperative recovery: a meta-analysis
Source: BMC Anesthesiol. 2024 Mar 19;24:104. doi: 10.1186/s12871-024-02483-z (PMC10949587; doi:10.1186/s12871-024-02483-z)
Supplement: Supplementary file 2 — Supplementary Material 2 [file 12871_2024_2483_MOESM2_ESM.docx]

**Supplementary** **document** **1:** The search strategy

**Pubmed** (https://pubmed.ncbi.nlm.nih.gov/)

Search: **(((transcutaneous acupoint electrical stimulation[Title/Abstract]) OR (transcutaneous electrical acupoint stimulation[Title/Abstract])) OR (electroacupuncture[Title/Abstract])) OR (electro-acupuncture[Title/Abstract])) OR (TEAS[Title/Abstract])** AND **((quality of recovery) OR (quality)) AND (recovery)**

**Embase** (http://www.embase.com/)

#1 'transcutaneous acupoint electrical stimulation'

#2 'transcutanclus electrical acupoint stimulation':ab,ti OR 'electroacupuncture':ab,ti OR 'electro-acupuncture':ab,ti OR 'TEAS':ab,ti

#3 #1 OR #2

#4 '**quality of recovery**'

#5 **quality**':ab,ti AND '**recovery**':ab,ti

#6 #4 OR #5

#7 #3 AND #6

**Web of Science** (http://apps.webofknowledge.com/)

#1 TS= (transcutaneous acupoint electrical stimulation OR transcutanclus electrical acupoint stimulation OR electroacupuncture OR electro-acupuncture OR TEAS)

#2 TS=( quality of recovery OR quality **OR** recovery)

#3 #1 and #2

**Cochrane** (https://www.cochranelibrary.com/)

#1 'transcutaneous acupoint electrical stimulation'

#2 (transcutaneous acupoint electrical stimulation):ti,ab,kw OR (transcutaneous electrical acupoint stimulation):ti,ab,kw OR (electroacupuncture):ti,ab,kw OR (electro-acupuncture):ti,ab,kw OR (TEAS):ti,ab,kw

#3 #1 OR #2

#4 (quality of recovery):ti,ab,kw

#5 (quality):ti,ab,kw

#6 (recovery):ti,ab,kw

#7 #5AND #6

#8 #4 OR #7

#9 #3 AND#8

**The search strategy used for CNKI and Wan Fang Database**

**（主题：经皮穴位电刺激）OR（主题：穴位电刺激） AND（主题：恢复质量）OR（主题：恢复）OR（主题：质量）**
