# Supplementary material for: Effect of transcutaneous electrical acupoint stimulation on the quality of postoperative recovery: a meta-analysis
Source: BMC Anesthesiol. 2024 Mar 19;24:104. doi: 10.1186/s12871-024-02483-z (PMC10949587; doi:10.1186/s12871-024-02483-z)
Supplement: Supplementary file 3 — Supplementary Material 3 [file 12871_2024_2483_MOESM3_ESM.docx]

**Supplementary** **document** **2:the Sensitivity analysis**

**Sensitivity analysis of 24-hour postoperative QoR-40 score**

**Sensitivity analysis of Emotional state**

**Sensitivity analysis of physical comfort**

**Sensitivity analysis of Physical independence**

**Sensitivity analysis of pain**

**Sensitivity analysis of 24-hour postoperative VAS pain score**
